# Social Contact Networks and Disease Eradicability under Voluntary Vaccination

**DOI:** 10.1371/journal.pcbi.1000280

**Published:** 2009-02-06

**Authors:** Ana Perisic, Chris T. Bauch

**Affiliations:** Department of Mathematics and Statistics, University of Guelph, Guelph, Ontario, Canada; University of Texas at Austin, United States of America

## Abstract

Certain theories suggest that it should be difficult or impossible to eradicate a vaccine-preventable disease under voluntary vaccination: Herd immunity implies that the individual incentive to vaccinate disappears at high coverage levels. Historically, there have been examples of declining coverage for vaccines, such as MMR vaccine and whole-cell pertussis vaccine, that are consistent with this theory. On the other hand, smallpox was globally eradicated by 1980 despite voluntary vaccination policies in many jurisdictions. Previous modeling studies of the interplay between disease dynamics and individual vaccinating behavior have assumed that infection is transmitted in a homogeneously mixing population. By comparison, here we simulate transmission of a vaccine-preventable SEIR infection through a random, static contact network. Individuals choose whether to vaccinate based on infection risks from neighbors, and based on vaccine risks. When neighborhood size is small, rational vaccinating behavior results in rapid containment of the infection through voluntary ring vaccination. As neighborhood size increases (while the average force of infection is held constant), a threshold is reached beyond which the infection can break through partially vaccinated rings, percolating through the whole population and resulting in considerable epidemic final sizes and a large number vaccinated. The former outcome represents convergence between individually and socially optimal outcomes, whereas the latter represents their divergence, as observed in most models of individual vaccinating behavior that assume homogeneous mixing. Similar effects are observed in an extended model using smallpox-specific natural history and transmissibility assumptions. This work illustrates the significant qualitative differences between behavior–infection dynamics in discrete contact-structured populations versus continuous unstructured populations. This work also shows how disease eradicability in populations where voluntary vaccination is the primary control mechanism may depend partly on whether the disease is transmissible only to a few close social contacts or to a larger subset of the population.

## Author Summary

Interest in infectious disease models that incorporate the effects of human behavior has been growing in recent years. However, most of these models predict that it should never be possible to eradicate a disease under voluntary vaccination, due to nonvaccinating “free riders” that emerge when vaccine coverage is high. This prediction contradicts the fact that smallpox was eradicated under a voluntary vaccination policy in many jurisdictions, and that other diseases such as polio are likewise near eradication. These previous models assumed that populations mix homogeneously. However, for some diseases, such as HIV and smallpox, individuals are more likely to get the disease from certain social contacts. Here we show that using a network model that captures this social structure can reconcile the previous theories to the empirical fact that diseases can be eradicated under voluntary vaccination. When infection is transmitted only through close contacts in the network, then an outbreak can be quickly contained using only voluntary vaccination. However, when infection can potentially be transmitted to almost everyone in the network (such as for measles), a disease outbreak can never be contained using voluntary vaccination. This latter observation may have some relevance to the Measles–Mumps–Rubella autism “vaccine scare.”

## Introduction

Model-based analyses of vaccination programmes have often concluded that it should be difficult or impossible to eradicate a vaccine-preventable disease under a voluntary vaccination policy without other incentives [1]–[10]. As vaccination coverage increases, the disease becomes increasingly rare due to herd immunity. Eventually, the infection risk to susceptible individuals decreases to zero, while the individual risk due to the vaccine remains constant. Hence, the individual motive to vaccinate is also reduced to zero as vaccine coverage increases. This should be true, in principle, even for a disease such as smallpox with very high case fatality rates, as long as the infection risk is deemed sufficiently small. This effect, similar to the “Prisoner's Dilemma” has also been explored in game theoretical analyses of infectious disease dynamics and vaccination [3]–[6],[8],[9]. In game theoretical treatments, it has been shown that vaccine coverage beyond the eradication threshold is not a Nash equilibrium if vaccine risk is nonzero, because a small group of individuals can achieve a higher payoff by switching to a nonvaccinator strategy [4]. Such strategic, self-interested behavior has been suggested as a possible contributor to vaccine scares in countries with a voluntary vaccination policy, such as England & Wales, which experienced declines in vaccine uptake for pertussis in the 1970s [4],[5],[11], and in measles–mumps–rubella (MMR) vaccine uptake more recently [12]. Recent work has explored exceptions to this rule, for instance finding cases of multiple equilibria when virulence varies with age [13] and when vaccines are sufficiently imperfect [14].

To date, smallpox is the only vaccine-preventable disease ever to have been globally eradicated [15], although polio is closer to eradication than ever before [16]. The last foothold of smallpox was in low-income countries, particularly in Africa and South Asia [17]. Jurisdictions in these countries often had widely varying vaccination policies. For instance, vaccination was compulsory in some Indian states, but voluntary in others [10]. Even in the final stages of eradication, when outbreaks were becoming less frequent, individuals often continued to voluntarily opt for vaccination, without the benefit of individual financial incentives to vaccinate. If the foregoing theories are correct that diseases cannot generally be eradicated under voluntary vaccination, how was smallpox globally eradicated despite voluntary vaccination in some jurisdictions?

Most, if not all, previous mathematical models that analyze discrepancies between individually and socially optimal vaccination strategies under voluntary vaccination have considered populations without spatial or social contact structure (e.g. social networks), and where populations are large enough for the continuum approximation to apply. This also appears to be true of behavior-infection models more generally [18], including those that study vaccine supply-demand dynamics at the international level, and non game-theoretical treatments of the problem. In these previous analyses, populations are generally considered to be homogeneously mixing, meaning that individuals are as likely to be infected by a member of their own household as they are by someone from the general public. However, the inadequacy of homogenous mixing models for certain situations has been widely documented, as have the differences between the predictions of homogeneous-mixing models and models where transmission is constrained to take place on a contact network [19]–[23]. In the present context, the homogeneous mixing assumption is arguably a good approximation for highly transmissible diseases spread primarily through aerosol droplets, such as measles. However, the assumption seems less valid for diseases that are transmitted through close contact, such as sexually-transmitted infections. Despite a few spectacular and widely reported cases of aerosol transmission [24], smallpox is also spread primarily through close contact, and typically requires prolonged face-to-face contact [25].

Here, we show that disease dynamics under a voluntary vaccination policy are substantially and qualitatively altered by the introduction of individual-level social contact structure. We analyze a social contact network model, where each node represents an individual, and each link represents a close contact through which infection may spread. Individuals decide whether or not to vaccinate based upon their expected payoffs for vaccinating versus not vaccinating. We assume the vaccine is free to individuals, which describes the situation for many major pediatric vaccines in advanced countries, as well as the situation under the WHO smallpox eradication program in the 1970s. We first study epidemics on this contact network for a general vaccine-preventable infection with simplified SEIR-type (Susceptible-Exposed-Infectious-Recovered) disease history. At baseline parameter values, for small neighborhood sizes, outbreaks are quickly contained using only voluntary ring vaccination. As the neighborhood size increases while infection risk (force of infection) is held constant, a threshold neighborhood size is reached. Above this threshold, voluntary vaccination fails and the population experiences both a considerable final epidemic size and a large number vaccinated. Hence, the limit of large neighborhood size in this model recovers dynamics similar to those of homogeneous mixing models. Because the force of infection is held constant as neighborhood size increases, the failure of voluntary vaccination is attributable solely to a decrease in how localized disease transmission is on the network. We associate smaller neighborhood sizes with close contact infections such as smallpox, and larger neighborhood sizes with diseases that do not require close contact for transmission, such as measles. We carry out a similar investigation for smallpox-specific disease history and vaccine properties, with similar results. This analysis illustrates the importance of considering discrete, contact-structured populations for modeling vaccinating behavior for close contact infections, and provides a framework for reconciling previous theoretical predictions concerning the ineradicability of infectious diseases under voluntary vaccination to the empirical fact of the global eradication of smallpox and local elimination of many other infectious diseases through voluntary vaccination.

## Methods

Here we describe the social contact network, infection transmission, and assumptions regarding individual vaccinating behavior. Definitions of parameters and corresponding parameter values appear in Table 1. We consider the case of a single epidemic outbreak. Because demographic processes are relatively slow on this timescale, we use a static network that does not include birth or death (other than due to the disease). An outbreak scenario was chosen, rather than an endemic situation, because the epidemiology of an infectious disease close to being eradicated is more aptly described in terms of stochastic outbreaks rather than sustained endemic transmission. The neighborhood size (node degree) of the network is Poisson-distributed with mean 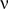. The network is formed by adding links to randomly selected nodes until the desired value of 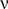 has been reached. After this, no further links are added, and the infection is introduced by inoculating 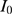 randomly chosen nodes out of a population of size 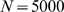 composed of susceptible individuals. We chose 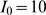 to ensure that outbreaks did not fail simply due to stochastic effects. Additionally, we ensured 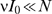 so that successful outbreaks could go through at least two generations before depleting the pool of susceptible individuals. Hence, we expect results to be similar on larger networks. There is a probability 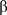 per day at which an infectious node infects a neighboring susceptible node. Hence, if a susceptible node has 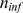 infectious neighbors on a given day, the total probability 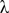 that the node becomes infected on that day is
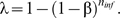
(1)The timestep of the simulation is one day, and each node's status is updated at the end of each day.

**Table 1:** Baseline parameter values for SEIR-type infection.

Parameter	Meaning	Value	Reference
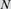	Population size	5,000	
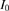	Initial number of individuals inoculated with smallpox	10	
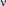	Mean neighborhood size	10	
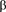	Probability of node-to-node transmission	0.02 day^−1^	[50]
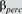	Perceived probability of node-to-node transmission	0.02 day^−1^	[50]
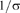	Mean duration of latent period	12 days	[51]
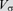	Variance of latent period	4 days^2^	[51]
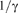	Mean duration of infectious period	19 days	[51]
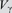	Variance of infectious period	4 days^2^	[51]
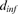	Probability of death due to infection	0.3	[52],[53]
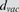	Probability of death due to vaccine-related complications	0.001	[36]
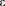	Vaccine efficacy	0.95	[50]
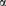	Payoff for individuals with continued susceptibility	40 life-years	[30]
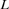	Payoff for individuals with lifelong immunity	40 life-years	[30]

On any given day, each individual who has not already vaccinated decides whether or not to vaccinate depending upon their perceived risk of infection from neighbors versus the perceived risks of vaccination. We assume that infectiousness coincides with the onset of symptoms, and that individuals base vaccination decisions upon the presence of symptoms in a neighbor. If an individual has 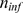 infected neighbors, we assume that the perceived probability 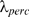 per day of being infected today is given by
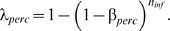
(2)where 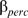 is the perceived probability per day that the individual is infected by a single given infectious neighbor. If the payoff 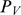 for vaccinating exceeds the payoff 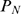 for not vaccinating, then the individual seeks and acquires vaccination on that day. If the individual does not vaccinate today, the individual can still vaccinate on following days.

### SEIR Infection

For the vaccine-preventable SEIR infectious disease, we assume that a newly-infected person remains in the latent stage for a duration of time drawn from a gamma probability density function (PDF) with a mean of 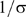 days and a variance of 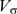 days^2^. After this time, the individual becomes infectious and remains so for a duration of time drawn from a gamma PDF with a mean of 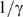 and a variance of 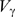 days^2^. Subsequently, the individual either dies with perceived probability 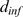 due to fatal disease complications, or recovers to lifelong immunity with perceived probability 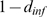. A gamma PDF was used for its convenience and realism [26]. The vaccine has efficacy 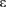 and is assumed to confer lifelong immunity. The currency of the payoff functions is the number of life-years that the individual can expect to accrue as a result of their strategy choice. We do not consider non-fatal outcomes such as long-term health conditions and we assume that the start of infectiousness coincides with the appearance of symptoms. We also assume a relatively high baseline perceived probability of death due to vaccine, 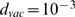, to reflect the fact that individuals often have inflated perceptions of vaccine risks [27],[28].

We first consider the payoff 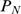 for not vaccinating today. If the individual does not vaccinate, s/he is either infected today (with perceived probability of infection 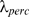) or not (with probability 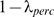). If the individual is infected today, then s/he experiences fatal disease complications with probability 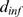, accruing no additional life years. If the individual survives (probability 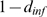), s/he can expect to accrue 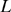 additional life years, where 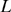 is the average remaining life expectancy for an individual who has lifelong immunity to the disease. Hence, the payoff if the nonvaccinating individual is infected is 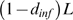. On the other hand, if the nonvaccinating individual escapes infection today, then s/he receives a payoff of *α*. This payoff represents the individual's expected remaining life years, given that they remain susceptible today because they were neither vaccinated nor infected. The total payoff 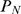 to an individual who does not vaccinate today is therefore
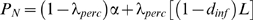
(3)


The parameter 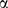 is influenced by the likelihood of being infected or of being compelled to vaccinate later on during the current epidemic oubreak in the population, and the likelihood that these result in death. It is impractical to derive a closed form expression for the probabilities of all these events in order to generate an expression for 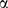. Also, in reality, individuals may resort to “rules of thumb” when weighing future outcomes. However, it is reasonable in any case to expect 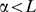, since a person with immunity can expect to outlive a person without immunity, on average.

Over time horizons longer than the current epidemic, other factors may contribute to what assumptions must be made about 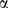. For instance, if vaccine-derived immunity wanes and the population is subject to another outbreak 20 years hence, then vaccination today may appear less favourable, even if 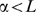 is still valid. However, these distant future events would be heavily discounted [29] and so we will restrict our time horizon to the current outbreak only.

Due to uncertainties in exactly how much 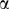 is less than 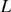, for our baseline analysis we assume 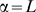 (though we explore 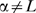 in sensitivity analysis). This makes nonvaccinating behavior more attractive and hence is a conservative assumption with respect to demonstrating the success of voluntary vaccination in containing outbreaks in a contact-structured population: if voluntary vaccination can control an oubreak for 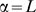, it should also be absent when 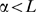. In the baseline scenario, we assume 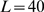 years, representing the average remaining life expectancy in good health in a typical developed country [30]. We note that the baseline value of 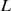 does not change the model dynamics qualitatively or quantitatively, since 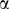 scales with 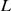 (subject to above assumptions about future outbreaks).

We next compute the payoff 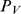 to vaccinate today. If a person chooses to vaccinate, then either the vaccine is efficacious (probability 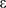), or not (probability 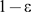). We suppose that the vaccine carries a perceived probability 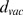 of death due to complications from the vaccine, where 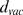 is generally small. (Under the assumption of perfect information, we note that 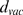 could also be interpreted as the empirical probability of death.) If the vaccine is efficacious but results in fatal complications, then no additional life years are accrued. However, if the vaccine is efficacious and no complications ensue, then the individual receives a payoff
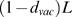
(4)


By comparison, if the vaccine is not efficacious, then either the individual dies from vaccine complications, accruing zero additional life years (probability 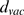), or the individual does not die (probability 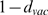). If the individual does not die from the vaccine, then either the individual is infected today, or the individual is not infected today. If the individual is not infected today (probability 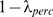), then the individual has escaped both death due to the vaccine and infection and remain susceptible, so their payoff will be 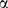. However, if the person is infected (probability 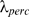), then they suffer a perceived probability 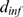 of dying due to the disease, but if they survive (probability 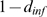), their payoff is lifelong immunity, or 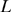 accrued life-years. Hence, in the case that the vaccine is not efficacious, the total payoff is
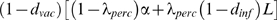
(5)and from Equations 4 and 5, we have
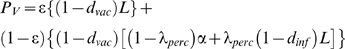
(6)


On any given day, if
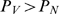
(7)then the individual decides to vaccinate. Otherwise they may still vaccinate in future according to the same decision rules, and with no influence from their previous decision history.

### Smallpox Infection

To tailor the model to the case of smallpox, we incorporate a more realistic description of the natural history of smallpox and the effects of vaccination. We incorporate the fact that conventional smallpox vaccine can work in individuals who are already infected: those who receive the vaccine within 3 days of infection usually do not become infectious and subsequently recover with long-term immunity, those who receive the vaccine 4–7 days post-exposure experience less severe symptoms but will be as infectious as an unvaccinated person, and those who receive the vaccine more than 7 days post-exposure gain no benefit [31]. Assumptions about network structure were the same as for the SEIR-type infection. For the case of smallpox, we used an empirical value of 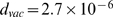 [32], though we discuss the effect of using larger values of 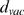.

To compute payoffs for the case of smallpox, we accounted for both fatal and non-fatal outcomes, such as pockmarks or blindness due to vaccine or disease. In order to compare fatal and non-fatal health outcomes in the same payoff function, the payoffs were expressed in terms of quality-adjusted life years (QALYs), i.e., the number of life years accrued, multiplied by a utility score between 0 (death) and 1 (perfect health) to reflect quality of life during those years [33]. Utilities for each possible health state after vaccination or smallpox infection were obtained from the literature. The payoff functions for the generic vaccine-preventable infection (Equations 3 and 6) were thus adjusted to obtain the payoff functions for the case of smallpox:
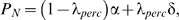
(8)

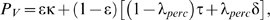
(9)where 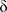 is the payoff to a person who becomes infected today by smallpox, 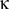 is the payoff to a person who vaccinates today and in whom the vaccine has been efficacious (but who may experience complications or death due to vaccine), and 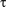 is the payoff to a person who vaccinates today and in whom the vaccine is not efficacious, but who avoids becoming infected today (and who may experience complications or death due to vaccine). Details of the derivation, as well as the full expressions for 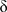, 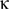, and 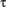 in terms of model parameters and utilities appear in Text S1. We note that 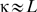, 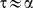, and 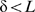 at realistic parameter values. Again we assume 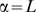 for our baseline scenario. Parameter values appear in Text S1. An equation analogous to Equation 10 can also be derived, with a similar interpretation.

## Results

It is possible to show from Equations 3 and 6 that an individual with at least one infectious neighbor will vaccinate (i.e., 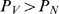 for the individual) if and only if
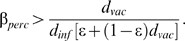
(10)


The derivation of this expression appears in Text S1. We note that 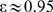 for many common vaccines. Also, 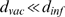 in most cases except when individuals have unrealistically inflated assessment of vaccine risks, such as during a “vaccine scare.” Therefore, whether or not an individual with at least one infectious neighbor vaccinates hinges largely upon the value of 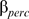. If 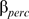 is sufficiently high then self-interested (rational) vaccinating behavior will result in eradication in a contact-structured population. (We note that rational behavior is precisely what causes failure to eradicate in a homogeneously-mixing population under a voluntary vaccination policy [4].) On the other hand, if 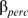 is sufficiently small, then an individual with at least one infectious neighbor may not judge the risk of infection great enough to vaccinate, and the infection may thereby spread through the entire population. We note that Equation 10 holds regardless of the assumed network structure.

Low values of 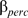 may occur for an infectious diseases where the number of social contacts through which infection can potentially be transmitted is large, but the probability of becoming infected from any single given contact is small. For instance, the number of potential effective contacts for measles transmission is large: the virus can be transmitted through aerosolized droplets that remain airborne for long periods, therefore the list of individuals to whom measles infection can potentially be passed is very large. However, even in a fully susceptible population, only 10–20 of these individuals are actually infected [34]. By comparison, we note that 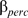 may be high for a sexually transmitted or close contact infection, where the list of contacts to which infection can be transmitted may be small, but the probability of transmission to each contact is high.

In the present model, as the average neighborhood size increases and the node-to-node transmission rate declines correspondingly, we should therefore expect to find conditions where Equation 10 is violated and voluntary vaccination fails to contain the outbreak, as predicted by models that assume homogeneous mixing. To test for this, we simulated the spread of the SEIR-type and smallpox infections on the network under a wide range of possible values for the average neighborhood size 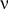. For each value of 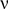 tested, the value of the node-to-node transmission rate 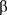 was adjusted so that the average force of infection per susceptible node remained the same as for the baseline parameter values. Holding the average force of infection constant as neighborhood size increases is crucial, since any change in model dynamics is thereby attributable to a decline in how localized the contact network is, rather than an increase in the overall force of infection (see [35] for an analogous approach for the effects of concurrency). Method I for maintaining constant infection risk assigned 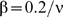 day^−1^ for each value of 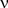 tested (where 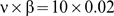 day^−1^ = 0.2 day^−1^ for the baseline parameters). Hence, a doubling of the number of social contacts would reduce the node-to-node transmission rate by half, which should maintain approximately the same infection risk per susceptible node as in the baseline scenario. However, higher-order effects relating to network structure are not accounted for in this approach and could change the infection risk in subtle ways. Therefore, we also controlled for infection risk using Method II, which involved calibrating the value of 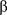 to ensure the measured infection risk was the same across all neighborhood sizes. For each value of 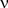, simulations were run for a range of finely stratified 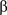 values. The value of 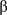 which yielded a final epidemic size within 0.2% of the average baseline final size of 10 realizations was selected. Hence, for each value of 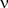, we know a corresponding value of 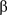 for which a given susceptible person has the same chance of becoming infected as for the baseline scenario. The simulations were run in a population without vaccination, since the goal was to obtain a range of infections with the same average risk to individuals but varying neighbourhood sizes, and including vaccination would thus bias this calibration. All other parameters were as in Table 1, except we maintained 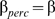 throughout.

**Figure 1:**
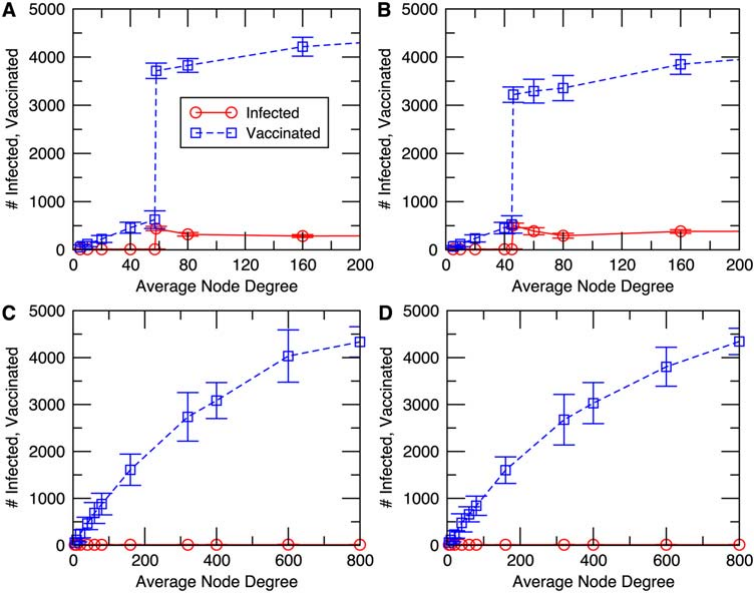
Final size and number vaccinated versus average neighborhood size for an SEIR-type infection, under Method I (A) and Method II (B), and for smallpox, Method I (C) and Method II (D). 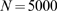
. Error bars represent two standard deviations from the mean across 20 simulations per datum.

For the SEIR-type infection, the simulation results under Method I indicate that voluntary vaccination contains the outbreak until the average neighborhood size 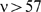 (Figure 1A). Below this threshold, both the final size and total number vaccinated remain small, since the outbreak is easily controlled through voluntary ring vaccination. Above this threshold, each infected node infects more than one neighboring node on average (usually a nonvaccinator with insufficient infectious neighbors to compel him/her to vaccinate). As a result, the infection breaks through imperfect rings of vaccinated individuals, and percolates through the entire network until almost all individuals have been either vaccinated or infected. This outcome is qualitatively similar to the divergence between individually and socially optimal strategies observed in homogeneous mixing models–these exhibit regimes where a large number of individuals can choose to vaccinate, but never enough to eradicate the disease completely [4]. The simulation results under Method II are very similar, with failure of voluntary vaccination materializing once 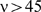 (Figure 1B). Hence, social contact structure can enable outbreak containment even when the neighborhood size is relatively large. We note that for 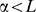, the threshold values of 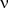 would be higher.

For smallpox infection, voluntary vaccination contains the outbreak for all 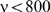 (Figure 1C and 1D). This difference compared to the SEIR-type infection is attributable mainly to the difference in the perceived probability of death due to the vaccine, 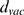. In the case of the SEIR-type infection, the perceived probability was taken to be a relatively high 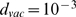, since individuals tend to inflate vaccine risks [36]. However, for smallpox, we used the empirical value 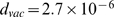. Therefore from Equation 10 we observe that value of 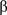 must be very low (i.e., 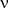 must be very large) before voluntary vaccination fails to contain the outbreak. This would require a simulated population size on the order of 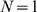 million, but our simulation became prohibitively slow beyond 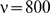. We note that homogeneous mixing models assume the continuum approximation holds, such that 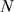 is a real number, and therefore voluntary vaccination should fail to contain an outbreak even for arbitrarily small vaccine risks and arbitrarily high case fatality rates. Simulations for smallpox with an inflated 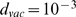 are similar to those for the SEIR-type infection, with voluntary vaccination not failing until the neighborhood size is significant.

**Figure 2:**
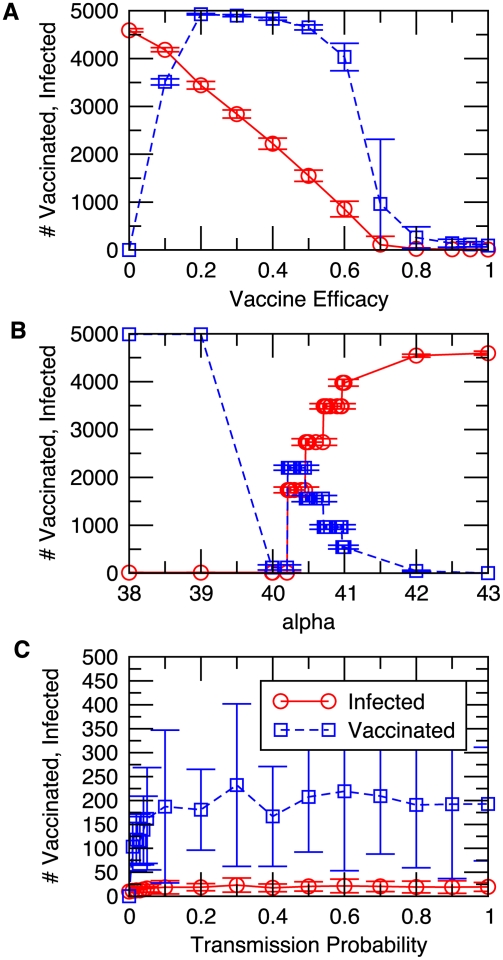
Final size and number vaccinated versus vaccine efficacy 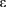 (A), future payoff under continued susceptibility 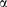 (B), and node-to-node transmission probability 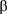 (C), for the SEIR-type infection. 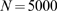
. Error bars represent two standard deviations from the mean across 20 simulations per datum. Note difference in vertical scale for (C).

We also carried out a univariate sensitivity analysis with respect to the payoff 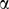 to individuals who remain susceptible, the node-to-node transmission rate 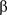, and the vaccine efficacy 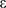. As each of these parameters was separately varied, the other parameters were kept at the baseline values. For the SEIR-type infection, in the case of varying 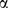, we observe that voluntary vaccination contains the outbreak for all 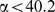 (Figure 2A). For 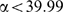, vaccination appears attractive even in the absence of an infected neighbor, and all individuals vaccinate. For 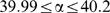, vaccination is attractive when there is one or more infectious neighbors, and the outbreak is controlled through voluntary ring vaccination. When 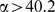, vaccination only becomes attractive once the number of infectious neighbors is already quite large (i.e., too large to prevent further transmission), and hence the final size is very large. As 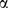 grows, the final size continues to grow and the number who seek vaccination continuously decline, since vaccination becomes progressively less attractive for larger 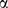. However, as discussed in the *Methods* section, biologically realistic solutions are restricted to 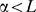. The final size and number vaccinated is not strongly dependent on the transmission rate 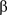 (Figure 2B). This is not surprising, since for small 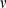 individuals rapidly vaccinate once it becomes known they have an infectious social contact, preventing further transmission. In the case of varying vaccine efficacy 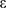 we observe that the final size increases monotonically as 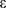 drop below 0.7 (Figure 2C). The number vaccinated is highest for 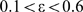. Below 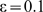, the number vaccinated is small because the vaccine is not efficacious enough to justify the individual decision to vaccinate. Above 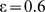, the number vaccinated is small because voluntary ring vaccination contains the outbreak relatively quickly. Results are similar for smallpox (Figure S1). We also tested the robustness of model predictions to several extensions, such as including delays in time required for the vaccine to confer immunity, probabilistic descriptions of the individual decision-making process, and the possibility that individuals may choose to vaccinate once their neighbor's neighbors become infected, but found no major qualitative differences in model dynamics at biologically realistic parameter values (results not shown).

Figures S2 and S3 depict several representative time series for the SEIR-type and smallpox infections respectively, at the baseline parameters values. As expected, secondary and tertiary transmission is limited in the baseline scenario, and occur only because of vaccine failures in some individuals. After each instance of secondary transmission, neighbors of the new case vaccinate and the outbreak is quickly controlled.

## Discussion

It is now a well-established principle in theoretical population biology that the dynamics of spatially structured populations can differ significantly from the dynamics of unstructured populations [37]–[39]. In spatially structured populations, coexistence, diversity and altruism develop more easily [40],[41], evolutionary velocities are slower [42], and epidemics have more realistic time series and critical community sizes [43]. Similar differences have been observed in the case of network models versus homogeneous mixing models [19]–[23]. The present model likewise illustrates dramatic changes in behavior-infection dynamics once social contact structure is introduced. When populations mix homogeneously, “rational” self-interested behavior leads to a divergence between individually and socially optimal strategies: vaccination coverage can be high, but never high enough to eradicate a vaccine-preventable infection. By comparison, the same kind of self-interested behavior in networks where contact structure is sufficiently local can rapidly and efficiently curtail outbreaks in discrete, contact-structured populations, through voluntary ring vaccination. This work also illustrates the importance of discrete effects in structured populations [37]. Events at the local, individual level (as played out in local neighborhoods on the network) can have significant implications for population-level outcomes such as final size and total number vaccinated. Finally, the present analysis recovers the homogeneous mixing case in the limit of large neighborhood size, thus capturing two qualitatively distinct regimes of behavior-infection dynamics. It thereby provides a framework for reconciling the predictions of behavior-infection models that assume homogeneously mixing populations to the empirical fact that many infectious diseases have been either globally eradicated or locally eliminated despite voluntary vaccination policies.

We expect that our results for SEIR infections should generally apply to vaccine-preventable infectious diseases where the SEIR disease history is a good approximation to the actual disease history, and where appearance of symptoms approximately coincides with the start of infectiousness. Examples of such diseases include smallpox (for the small neighbourhood limit), and measles, rubella, chickenpox, perhaps pertussis (for the large neighbourhood limit).

The basic reproductive number, 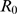 (the average number of secondary infections produced by a single infectious person in an otherwise susceptible population), is a fundamental measure that indicates how easily an infectious disease outbreak can be controlled. However, recent theory points to other aspects of infectious diseases that may be equally important. For instance, one such aspect is the proportion of transmission occurring before the onset of clinical symptoms [44]. Likewise, the current analysis suggests that whether a disease requires close contact for transmission may be an important factor determining the feasibility of outbreak control under voluntary vaccination policies.

Some previous game theoretical models have suggested that eradication is less likely to occur under voluntary vaccination when 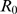 is higher, such as for measles and pertussis [4],[5]. The results from the present analysis dovetail with these earlier predictions, since diseases with higher 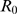 are also more likely to be spread through means other than close personal contact. For these more transmissible infections, the average neighborhood size is higher since the number of social contacts that could potentially result in transmission events is greater. Hence, the results of the present study also suggest that diseases with higher 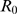 are more likely to be associated with failure to eradicate under voluntary vaccination. The present results also extend previous predictions by showing how the severity of the disease, as expressed through 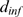, can influence whether voluntary vaccination will work.

It has previously been observed that spatial structure can also encourage the persistence of cooperative behavior in classical games such as the Prisoner's Dilemma [45]. This is phenomenologically similar to what occurs in the present study: in a contact-structured population, what is optimal for the group becomes coincident with what is optimal for the individual, when neighborhood size is small enough. In the case of the Prisoner's Dilemma, small clusters of cooperating individuals can maintain “equal footing” with clusters of defectors, because of the high cooperate-cooperate payoffs that occur within cooperator clusters. In the present analysis the mechanism is quite different, having to do with the large difference in infection risk between those who neighbor infectious individuals and those who do not. This individual-level structure cannot be described in a homogeneously mixing population subject to the continuum approximation [37].

There were several limitations to the present analysis. First, we assumed that 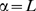. For a more realistic assumption of 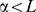, we would expect a higher threshold in neighborhood size for failure of voluntary vaccination. In the same vein, the payoff functions are static and do not reflect expected future disease dynamics. Static functions were used because we assumed that individuals do not weigh future outcomes heavily in the face of a current outbreak, especially if there are significant uncertainties about future disease dynamics. However, if individuals did make strategic choices according to expected future disease dynamics, the predictions may change. A second limitation is that we used a static, random network. Outcomes may differ for other types of networks. For instance, in a network with small characteristic path length [46], infection and vaccination should percolate through the network more quickly than was the case here, although we do not expect the final state of the network to change. Similarly, networks with scale-free neighborhood size distributions [47] may exhibit highly variable outcomes due to the large heterogeneity in neighborhood size: an infectious node with many neighbors could lead to a higher epidemic final size in the whole population, whereas a vaccinated node with few neighbors could significantly reduce final size. Finally, if network edges can form and be dissolved, then failure of voluntary vaccination should emerge for a sufficiently high rate of turnover of network edges, regardless of neighborhood size. Future work on behavior-infection dynamics in contact-structured populations should take into account more realistic networks such as those derived from household sizes, sexual behavior surveys, or contact-dairy studies. A third limitation is our assumption that becoming infective coincides with the appearance of recognizable symptoms. For diseases where individuals can transmit before exhibiting symptoms, voluntary ring vaccination would be less successful and we would expect the thresholds of Figure 1 to shift to the left. This is because individuals may still become infected if they wait to vaccinate until their neighbors exhibit symptoms. For smallpox in particular, some transmission may occur during the prodrome phase of infection, where individuals exhibit non-specific symptoms. However, because the contribution from this phase is relatively small [48] and smallpox has a low 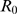, we do not expect this to qualitatively change our results. A fourth limitation is that we assumed that the vaccine works immediately upon innoculation. This is plausible for smallpox, since vaccination can prevent both disease and viral shedding in individuals if they are vaccinated within three days after becoming infected [31]. However, if a vaccine requires a sufficiently long time to take effect, waiting to vaccinate until a neighbor exhibits symptoms again may not prevent infection (though it may encourage individuals to vaccinate pre-emptively once disease has entered their local population). Models that accommodate these alternative possibilities could yield different predictions, and it is possible to imagine scenarios of disease propagation through individual-based networks that are analogous to the “race to trace” that has been described for universal smallpox vaccination [49]. Finally, we made the standard game theoretic assumption of “rational” behavior, although humans do not always act in this way. In this context, one implication is that individuals may react differently to outbreaks of a disease that has caused significant mortality very recently, compared to one that has not caused significant mortality in a long time.

Subject to the above limitations, there are *a priori* reasons to think that the basic dynamics illustrated here may describe a wide range of vaccine-preventable diseases that are spread through close contact. At the initial stages of an outbreak, the average prevalence of the disease across the whole population is very low. In a homogeneously-mixing population where infected persons are equally likely to make contacts with anyone in the population, the infection risk to any given individual is therefore also initially small, and individuals do not immediately seek vaccination. The disease thus transmits freely until the average prevalence is high enough to justify vaccinating for the remaining susceptible individuals. On the other hand, in a socially or spatially structured population, the short-term infection risk is heterogeneous across the population: nonexistent for most individuals, but very high for the immediate neighbors of an infected person. Hence, the neighbors of an infected person vaccinate as soon as their neighbor's symptoms appear. Since these effects are likely to apply to a broad range of infections spread through close contact, behavior-infection models of such infections may need to weigh the potential effects of individual-level contact structure.

## Supporting Information

Text S1 Additional details on mathematical derivations and smallpox parameter values.
(0.10 MB PDF)

Figure S1 Dependence of final epidemic size and final number vaccinated on vaccine efficacy epsilon (A), payoff alpha for continued susceptibility (B), and node-to-node transmission probability beta per day (C) for smallpox infection. *N* = 5000. Error bars represent two standard deviations from the mean across 20 simulations per data point.
(0.06 MB PDF)

Figure S2 Representative realizations from the simulation: time series of number infected and number vaccinated for the SEIR-type infection. *N* = 5000. Error bars represent two standard deviations from the mean across 20 simulations per data point.
(0.15 MB JPG)

Figure S3 Representative realizations from the simulation: time series of number infected and number vaccinated for smallpox infection. *N* = 5000. Error bars represent two standard deviations from the mean across 20 simulations per data point.
(0.15 MB JPG)
