# Supplementary material for: Social Contact Networks and Disease Eradicability under Voluntary Vaccination
Source: PLoS Comput Biol. 2009 Feb 6;5(2):e1000280. doi: 10.1371/journal.pcbi.1000280 (PMC2625434; doi:10.1371/journal.pcbi.1000280)
Supplement: Text S1 — Additional details on mathematical derivations and smallpox parameter values. (0.10 MB PDF) [file pcbi.1000280.s001.pdf]

Supporting Text

Derivation of payoff functions for smallpox

Here we derive the payoff functions for smallpox, equations (8) and (9). We first derive the payoff function for a nonvaccinator. A person who can expect not to contract smallpox for the rest of their life, due to their immunity from the vaccine or the disease, and who did not experience long-term complications due to vaccine or disease, accrues $L = 40$ QALYs, since they have approximately 40 years of life in perfect health left. A person who chooses not to vaccinate today is either infected today, or not infected today. If the nonvaccinator is infected, s/he receives a payoff

$$\begin{aligned} \delta &= (1 - d_{inf})\big[(1 - c_{dis,poc} - c_{dis,bli} - c_{dis,enc} - c_{dis,art}) \\ &\quad + c_{dis,poc}U_{poc} + c_{dis,bli}U_{bli} + c_{dis,enc}U_{enc} + c_{dis,art}U_{art}\big] L \end{aligned} \tag{11}$$

(see Table 3 for parameter definitions).

A person who remains susceptible today has an expectation of accruing α QALYs, where we again make the conservative assumption that $\alpha = L$ in the baseline scenario, even though we generally expect that $\alpha < L$. If a nonvaccinating individual is not infected today, their payoff should be α. Therefore, the total payoff for an individual who does not vaccinate today, according to their probability of being infected or not, is

$$P_N = (1 - \lambda_{perc})\alpha + \lambda_{perc}\delta. \tag{12}$$

On the other hand, if a person chooses to vaccinate today, the vaccine is either efficacious or not. If efficacious, then the individual receives the payoff

$$\begin{aligned} \kappa &= (1 - d_{vac})\left[(1 - c_{vac,ecz} - c_{vac,enc} - c_{vac,vac}) \\ &\quad + c_{vac,ecz}U_{ecz} + c_{vac,enc}U_{enc} + c_{vac,vac}U_{vac}\right] L \end{aligned} \tag{13}$$

If the vaccine is not efficacious, then the individual is either infected today or not, and either suffers vaccine complications or not. Because the disutility for being infected today

greatly outweighs the disutility for suffering vaccine complications, we restrict ourselves only the cases where (1) the person is infected today (the possibility of vaccine complications is neglected), and (2) the person is not infected today, but may or may not experience vaccine complications. The payoffs for these two cases are, respectively, δ and

$$\begin{aligned}\tau &= (1-d_{vac})\left[(1-c_{vac,ecz}-c_{vac,enc}-c_{vac,vac})\right.\\ &\quad \left.+c_{vac,ecz}U_{ecz}+c_{vac,enc}U_{enc}+c_{vac,vac}U_{vac}\right]\alpha \end{aligned} \tag{14}$$

where we note that $\tau = \kappa\alpha/L$ since the only difference in the two cases is whether or not the vaccine was efficacious. Hence, the payoff for the case where the vaccine is not efficacious is

$$\lambda_{perc}\delta+(1-\lambda_{perc})\tau \tag{15}$$

and the total payoff for a person who chooses to vaccinate today is therefore

$$P_V = \varepsilon\kappa+(1-\varepsilon)\left[\lambda_{perc}\delta+(1-\lambda_{perc})\tau\right]. \tag{16}$$

Parameter	Meaning	Value	Reference
N	Population size	5000	n/a
I_0	Initial number of individuals inoculated with smallpox	10	n/a
ν	Mean neighborhood size	10	n/a
β	Probability of transmission	0.027 day^{-1}	Ref. [50]
β_{perc}	Perceived probability of transmission	0.027 day^{-1}	Ref. [50]
$1/\sigma$	Mean duration of latent period	12 days	Ref. [51]
V_σ	Variance of latent period	4 days^2	Ref. [51]
$1/\gamma$	Mean duration of infectious period	19 days	Ref. [51]
V_γ	Variance of infectious period	4 days^2	Ref. [51]
d_{inf}	Probability of death due to smallpox	0.3	Ref. [52]
$c_{dis,poc}$	Probability of pockmarks in smallpox survivors	0.73	Ref. [53]
$c_{dis,bli}$	Probability of blindness in smallpox survivors	0.01	Ref. [53]
$c_{dis,enc}$	Probability of encephalitis in smallpox survivors	0.01	Ref. [53]
$c_{dis,art}$	Probability of arthritis in smallpox survivors	0.02	Ref. [53]
U_{poc}	Utility of a person with pockmarks due to smallpox	0.745	Ref. [32]
U_{bli}	Utility of a person with blindness due to smallpox	0.45	Ref. [32]
U_{enc}	Utility of a person with long-term encephalitis due to smallpox	0.666	Ref. [32]
U_{art}	Utility of a person with long-term arthritis due to smallpox	0.80	Ref. [32]
d_{vac}	Probability of death due to vaccine	0.27×10^{-5}	Ref. [32]
$c_{vac,ecz}$	Probability of long-term eczema due to the vaccine	3.80×10^{-5}	Ref. [32]
$c_{vac,enc}$	Probability of long-term encephalitis due to the vaccine	1.25×10^{-5}	Ref. [32]
$c_{vac,vac}$	Probability of progressive vaccinia due to the vaccine	0.15×10^{-5}	Ref. [32]
U_{ecz}	Utility of a person with long-term eczema due to the vaccine	0.745	Ref. [32]
U_{enc}	Utility of a person with long-term encephalitis due to the vaccine	0.666	Ref. [32]
U_{vac}	Utility of a person with progressive vaccinia due to the vaccine	0.35	Ref. [32]
ε	Vaccine efficacy	0.95	Ref. [50]
α	Payoff to a person with continued susceptibility	40 QALYs	Ref. [30]
L	Payoff to a person with lifelong immunity	40 QALYs	Ref. [30]

Table 2: Baseline parameter values for smallpox.

Parameter	Meaning
P_V	Payoff to a person who vaccinates today
P_N	Payoff to a person who does not vaccinate today
α	Payoff to a person who remains susceptible today
L	Payoff to a person who has acquired immunity, either through vaccine or through infection, and who did not experience long-term complications from vaccine or infection
δ	Payoff to a person who becomes infected today with smallpox
κ	Payoff to a person in whom the vaccine is efficacious (but who may experience vaccine complications)
τ	Payoff to a person in whom the vaccine is not efficacious, and who is not infected today (but who may experience vaccine complications)

Table 3: Payoff definitions used in derivation of payoff functions for smallpox

Derivation of Control Condition, Equation (10)

If an individual has no infectious neighbors ($\lambda_{perc} = 0$), then for $\alpha = L$ we have from Equations (3) and (6) that

$$P_V = (1 - d_{vac})\alpha < \alpha = P_N \tag{17}$$

Therefore, the individual will not choose to vaccinate: there is no perceived immediate risk of infection, so there is no need to accept even a small risk associated with vaccination. (In practice, individuals may choose to vaccinate as soon as they know an infection is present in their local population, and this would be consistent with successful outbreak control through voluntary vaccination.) However, if the individual has 1 infected neighbor, then $\lambda_{perc} = \beta_{perc} = 0.02$/day and we have that

$$P_V = L(1 - d_{vac})\left[1 - (1 - \varepsilon)\beta_{perc} d_{inf}\right] \tag{18}$$

$$P_N = L(1 - \beta_{perc} d_{inf}) \tag{19}$$

and it can be shown that $P_V > P_N$ if and only if

$$\beta_{perc} > \frac{d_{vac}}{d_{inf}\left[\varepsilon + (1-\varepsilon)d_{vac}\right]}\,. \tag{20}$$

Hence, equation (10) of the main text is obtained.
