# Supplementary figures and images for: Social Contact Networks and Disease Eradicability under Voluntary Vaccination
Source: PLoS Comput Biol. 2009 Feb 6;5(2):e1000280. doi: 10.1371/journal.pcbi.1000280 (PMC2625434; doi:10.1371/journal.pcbi.1000280)

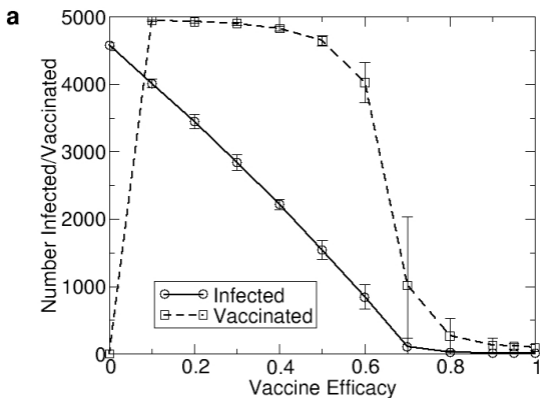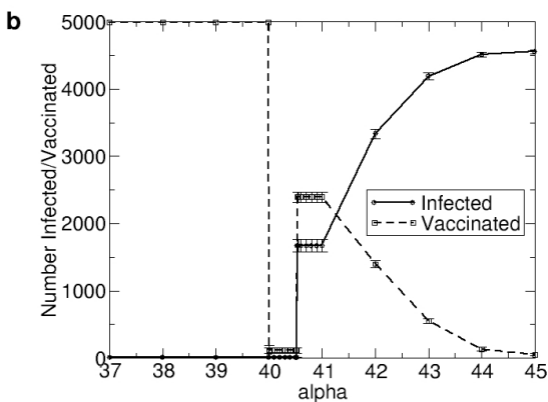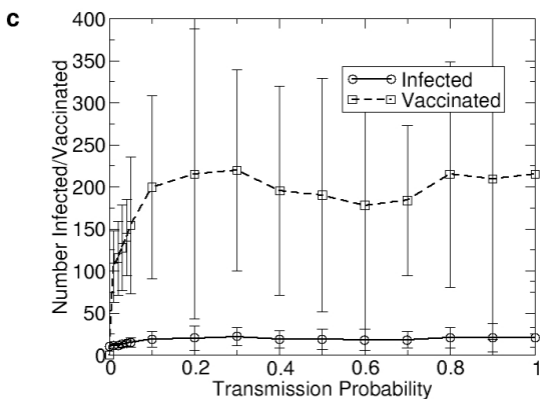

Supplement: Figure S1 — Dependence of final epidemic size and final number vaccinated on vaccine efficacy epsilon (A), payoff alpha for continued susceptibility (B), and node-to-node transmission probability beta per day (C) for smallpox infection. N = 5000. Error bars represent two standard deviations from the mean across 20 simulations per data point. (0.06 MB PDF) [file pcbi.1000280.s002.pdf]

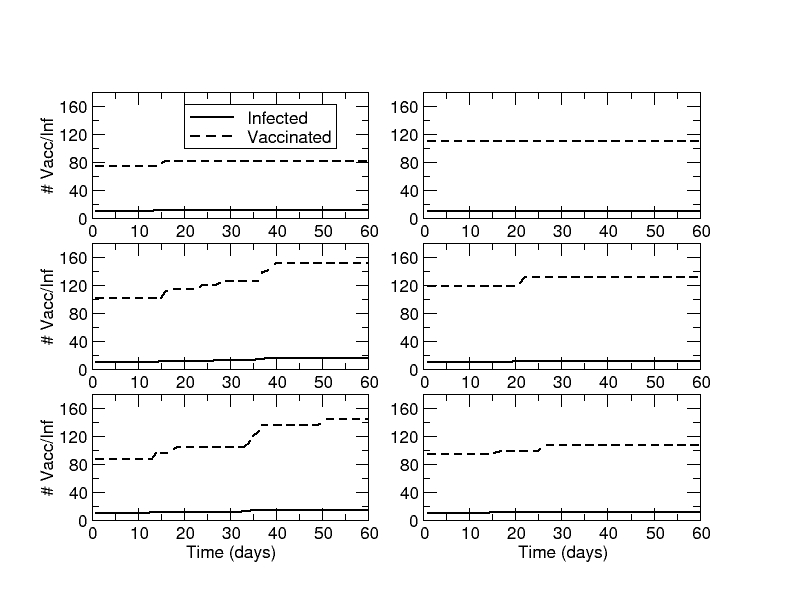

Supplement: Figure S2 — Representative realizations from the simulation: time series of number infected and number vaccinated for the SEIR-type infection. N = 5000. Error bars represent two standard deviations from the mean across 20 simulations per data point. (0.15 MB JPG) [file pcbi.1000280.s003.jpg]

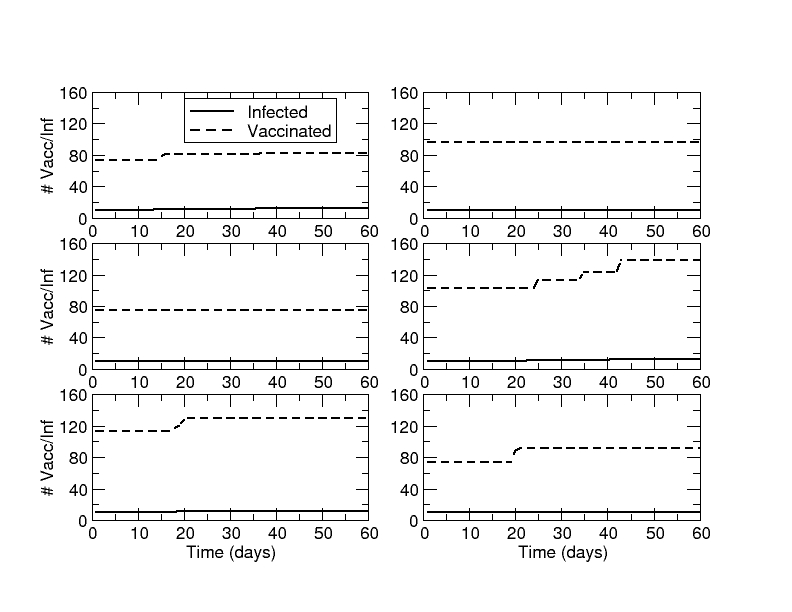

Supplement: Figure S3 — Representative realizations from the simulation: time series of number infected and number vaccinated for smallpox infection. N = 5000. Error bars represent two standard deviations from the mean across 20 simulations per data point. (0.15 MB JPG) [file pcbi.1000280.s004.jpg]
